# The Use of Infrared Thermography (IRT) as Stress Indicator in Horses Trained for Endurance: A Pilot Study

**DOI:** 10.3390/ani9030084

**Published:** 2019-03-07

**Authors:** Veronica Redaelli, Fabio Luzi, Silvia Mazzola, Gaia Dominique Bariffi, Martina Zappaterra, Leonardo Nanni Costa, Barbara Padalino

**Affiliations:** 1 Department of Veterinary Medicine (DIMEVET), University of Milan, 20133 Milano, Italy; veronica.redaelli@unimi.it (V.R.); fabio.luzi@unimi.it (F.L.); silvia.mazzola@unimi.it (S.M.); gaia.bariffi@libero.it (G.D.B.); 2 Department of Agricultural and Food Science (DISTAL), University of Bologna, 40127 Bologna, Italy; martina.zappaterra2@unibo.it (M.Z.); leonardo.nannicosta@unibo.it (L.N.C.); 3 Department of Veterinary Medicine, University of Bari, 70010 Bari, Italy

**Keywords:** thermography, training, endurance, horse

## Abstract

**Simple Summary:**

The aim of this study was to evaluate the possibility of using infrared thermography technique (IRT) to detect physiological stress in endurance horses. Infrared thermography technique, serum cortisol, blood count, and hearth rate (HR) were used as stress-indicators before and after training of different intensities (low, moderate, and high). As expected, all the studied parameters increased after the training, but HR remained below 64 bpm as requested by Fédération Equestre Internationale (FEI) endurance rules. Heart rate and white blood cells were the only parameters which significantly rose with the training intensity. Max temperature in the left and right eye (ET) was positively and significantly correlated with HR. These preliminary findings suggest that the tested horses were sufficiently fit and IRT may be a useful non-invasive tool to assess physiological stress in endurance horses.

**Abstract:**

The aim of this pilot study was to document the effects of endurance training at different intensities on heart rate (HR), blood count, serum cortisol, and maximal temperatures of different body locations, namely eye, crown, pastern pasterns, *gluteus* and *longissimus dorsi* muscle (*mm)*, measured by infrared thermography technique (IRT) in horses trained for endurance. Possible associations among the studied parameters were also investigated. Our hypothesis was that temperature, measured by IRT after endurance training of different intensities would vary depending on the intensity and would be positively correlated with HR and serum cortisol. Eight horses were tested before and after training of different intensities (low, moderate, and high). The results partially supported our hypothesis; all the studied parameters increased after training (*p* < 0.05), eye temperature (ET) correlated positively with HR (*p* < 0.01), and crown temperature (CT) correlated positively with cortisol (*p* < 0.01). However, only HR and white blood cells increased with the intensity of the exercise (*p* = 0.0016 and *p* = 0.0142, respectively). Our findings suggest the evaluation of ET and CT may become a useful non-invasive tool to detect physiological stress during training and to evaluate how the horses cope with the training. Infrared thermography technique may also become a useful tool for the early identification of horses that are not fit to compete or to continue the competition. However, further studies should be conducted on a larger number of horses and during competitions to ascertain our preliminary findings.

## 1. Introduction

Endurance is a rapidly growing equestrian discipline whose events have increased by 95% from 2007 to 2016 [1]. Endurance rides can be any distance, ranging from 40 to 160 km in a 24-h period, requiring horses to perform long-term exercise. To ensure the health and welfare of the endurance horses, designated veterinarians carry out health assessments of the horses at fixed distances (usually every 15 to 25 km) [1]. The health check typically includes the measurement of the following variables: heart rate, respiratory rate, rectal temperature, capillary refilling time, gut sounds, cardiac recovery index, and lameness scoring. Only the horses that are still apparently healthy and fit are allowed to continue to compete [2]. Endurance horses are commonly withdrawn due to three reasons: lameness, other medical problems, and overtime (i.e., the horse took too long to cover the distance). Colic, diaphragmatic flutters, exertional rhabdomyolysis, and heat exhaustion are among the most common medical problems observed in horses that have been pushed past their physiological limits [3]. Consequently, one of the roles of the veterinarians is to identify those horses under physical stress caused by the endurance competition and withdraw them before substantial injuries occur. However, it is well known that during a competition, horses are not only under physical but also under psychological/emotional stress [4], the latter leading to anxious behaviors and sweating, which may result in significant health problems. This is the reason why veterinarians should assess not only the physical but also the emotional state of the horses to decide whether they are fit to continue to compete, to safeguard the welfare of the endurance horses during competitions.

During endurance competitions, it is important to use non-invasive tools to assess the fitness of the horses. In fact, only non-invasive tools are allowed by Fédération Equestre Internationale (FEI) regulation [1] and endurance riders would not agree with the use of invasive tools, which may affect performance during competitions. Indeed, it has been reported that endurance riders mainly tend to use non-invasive tools, such as heart rate monitors with Global Positioning System (GPS) to check the fitness of their horses during training [5]. Infrared thermography (IRT) is a non-contact, non-invasive modality capable of detecting heat emitted from a body surface as infrared radiation [6]. Over the last thirty years, IRT has been widely used in veterinary medicine to detect injury, inflammatory responses, and causes of lameness, such as laminitis [7,8]. While the effective use of IRT as a tool to identify early pathologies, such as laminitis, is still a matter of debate [9], and IRT has been used to identify physiological stress in many species, including horses [10,11,12,13]. In particular, the evaluation of eye temperature (ET) seems very reliable [14,15,16]. Eye temperature was successfully used for assessing stress in horses and showed positive correlations with other stress indicators, such as heart rate (HR), heart rate variability (HRV), stress related behaviors, and cortisol [14,16,17]. Eye temperature was also successfully used to measure fear-related reactions of horses during a novelty test [18]. More recently, ET was proposed for the selection of horses intended for dressage competitions [19]. In particular, Sánchez et al. (2016) measured ET heritability and its correlation with performance and suggested that animals displaying higher ET after competitions should be not selected. Similarly, Negro et al. (2018) suggested that higher ET before racing (i.e., the horses were highly stressed before the competitions) could predict poor performance in Spanish trotter horses [4]. Infrared thermography technique has been used to detect performance-enhancing techniques in endurance horses [6]. However, to the best of the authors’ knowledge, the use of IRT, at the eye and in other body regions, has not been investigated to detect physiological stress in horses trained for endurance.

The hypothesis of this pilot study was that maximal temperature measured with IRT during endurance training of different intensities would vary depending on the intensity and would be positively correlated with heart rate and cortisol. The aim of this pilot study was to document the effects of training at different intensities on heart rate, blood count, cortisol, maximal temperature of different regions, namely eye, crown, pastern, *gluteus*, and *longissimus dorsi* measured with IRT in endurance horses. Possible associations among the studied parameters were also investigated. The findings may be useful to evaluate the possibility of using the infrared thermography technique (IRT) to detect the work-related stress responses during training and competitions in endurance horses.

## 2. Materials and Methods

### 2.1. Experimental Protocol

Experimental procedures, which involved the use of horses, were explained to owners and written informed consent was obtained before the experiment began. The procedures complied with the European Code of Practice for the care and use of animals for scientific purpose (DL n. 116, 27/01/1992), were considered as low risk and did not require approval by the Italian Ministry.

The trial took place at the horse stable Società Agricola “La Bosana”, Piozzano, Piacenza, Italy, which is an Arabian horse breeding and training center. All horses presented in the stable were scanned with the thermography unit prior to the initiation of the study and only those with no abnormal thermal patterns or asymmetries were included (*n* = 8). Table 1 shows the birth year, sex, coat, and number of previous competitions of the horses used in the study. 

**Figure 1:**
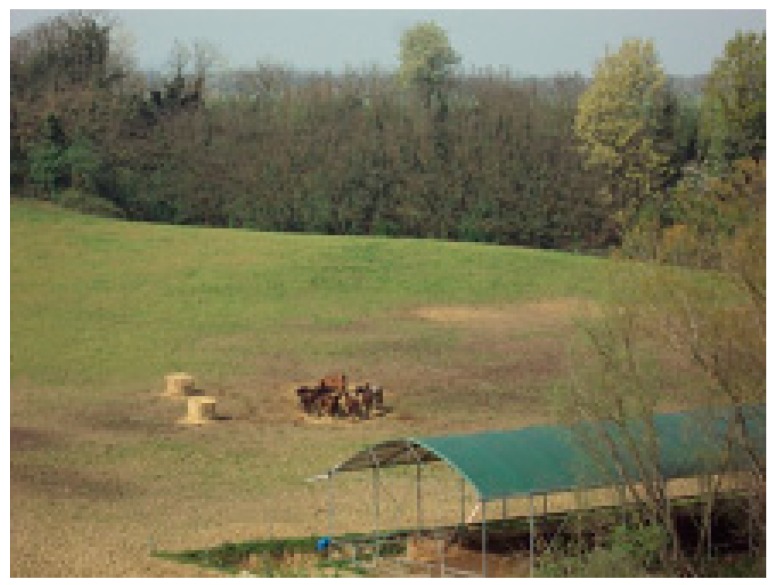
Paddock where the horses used for the experiment were kept daily and night.

The horses tested were Arabians born at “La Bosana” and were well accustomed to the horse stable management and training procedures carried out at the center. The horses were kept on pasture in large paddocks with shelters (Figure 1) all day long. They had lucerne hay and water ad libitum and were fed concentrate (commercial mix containing oats, corn, and barley) thrice a day (08:00; 12:00, and 18:00). However, feed ration was estimated individually in order to fulfill the animals’ individual needs.

**Figure 2:**
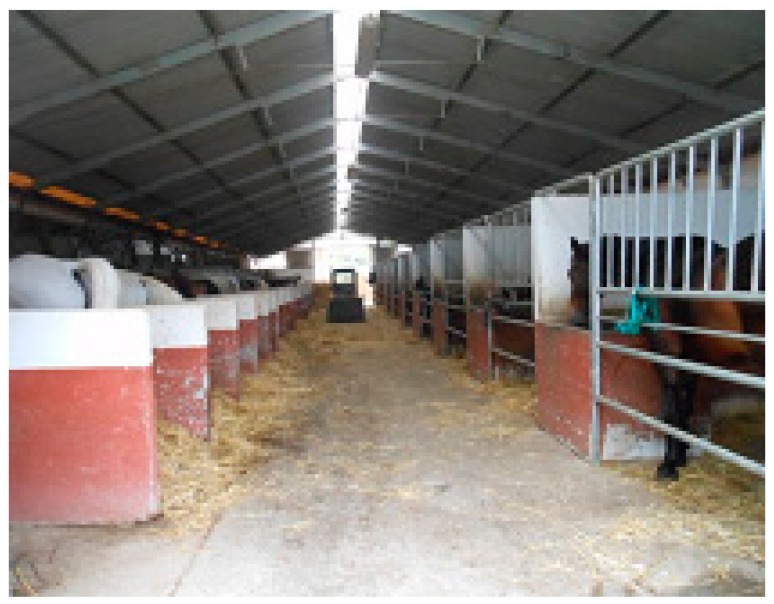
Horse stable where the horses were led and fed before the training.

The small morning meal of concentrated feed was given in the stable (Figure 2), where the horses were led to be fed and prepared for the training. The latter usually started around 09:00. After training, the horses were put back on pasture.

**Figure 3:**
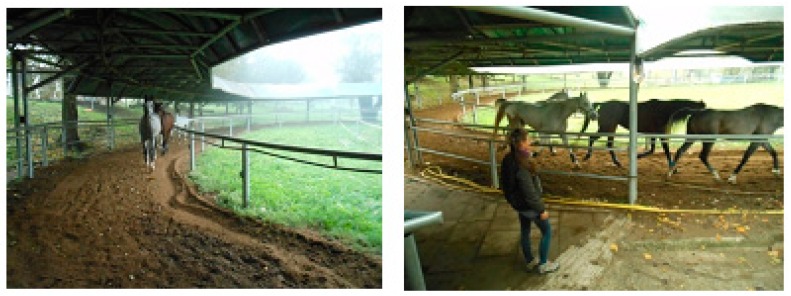
Walker where the horses were trained.

Over the three months before the start of the trial, the horses were subjected to the same training program. In particular they were being prepared for an International Endurance Race (90 km) and the trial was carried out once a week during the three weeks before the competition. The horses were exercised in a walker/horse training machine, where the horses could be trained without being ridden at different speeds and times automatically. The walker machine was a track of 160 m, all covered (Figure S1) and had two inner mechanical arms/panels which encouraged/controlled the horse to move at the desired speed. The panels hanged from a roller-coaster attached at the roof of the walker machine, and the horses were free to run between the two panels. The speed, duration, and direction of the training sessions were previously set at an external control panel, which controlled the mechanical arms/panels. Consequently, the horses were not in direct sunlight during the training sessions (Figure 3). Each training session started with a warm up (walk and slow trot for 5 min), followed by a planned training session, which was split into two parts to make the horses exercise clockwise and counterclockwise. All horses were well accustomed to this training, since they were trained in the horse walker using this procedure since they were yearlings. The owner reported that horses tended to show avoidance behaviors only at the beginning of the taming, and they quickly learn to gallop at the desired speed without being pushed or pulled by the mechanical arms.

**Figure 4:**
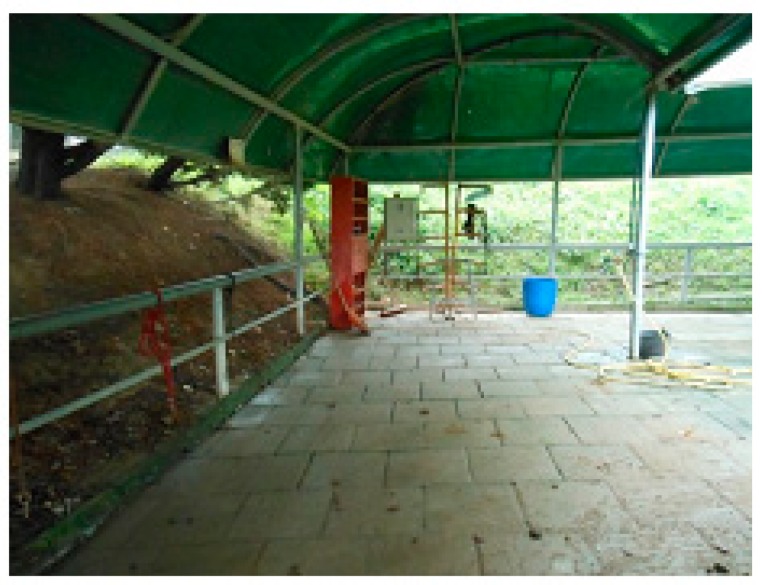
Area adjacent to the walker where the horses were cross tied and blood sampling, heart rate measurement, and infrared thermography technique (IRT) were performed.

The eight horses were split into two subgroups of four subjects which were always trained together at the three different intensities. The groups were formed according to the groups formed by the horses at pasture. The social groups were kept the same during the training in order to avoid any social stress and possible fighting, since the horses were trained freely. Based on the social herd hierarchy, during the training there was usually one leading mare followed by the other lower-ranking horses or sometimes the most fit horse was leading (see Figure 3). The training order of the subgroups were alternated across the three different training intensities, trying to take blood samples from all horses at the same time to avoid differences caused by circadian rhythms. During the trial, the horses were checked immediately before and after the following three training sessions: (a) light training (1 h at an average speed of 19 km/h) (low intensity); (b) medium training (2 h at an average speed of 16 km/h) (moderate intensity); and (c) strenuous training (3 h at an average speed of 20 km/h) (high intensity). Intensity, speed, and duration of the training sessions were chosen by the trainer/owners of the horses based on their experience. The horses were free to choose the gait (trot or gallop) to maintain the speed of the mechanical horse walker, but they tended to move at a constant speed gallop. The authors decided to keep this type of training to evaluate their effects without inducing any changes in the horse routine and because they considered this type of training unique and worth investigating, because in endurance the ability of the horse to self-regulate is considered as an important skill [1]. Before training (BT) and after training (AT) the horses were cross-tied in a cover area adjacent to the walker (Figure 4) to be checked.

**Figure 5:**
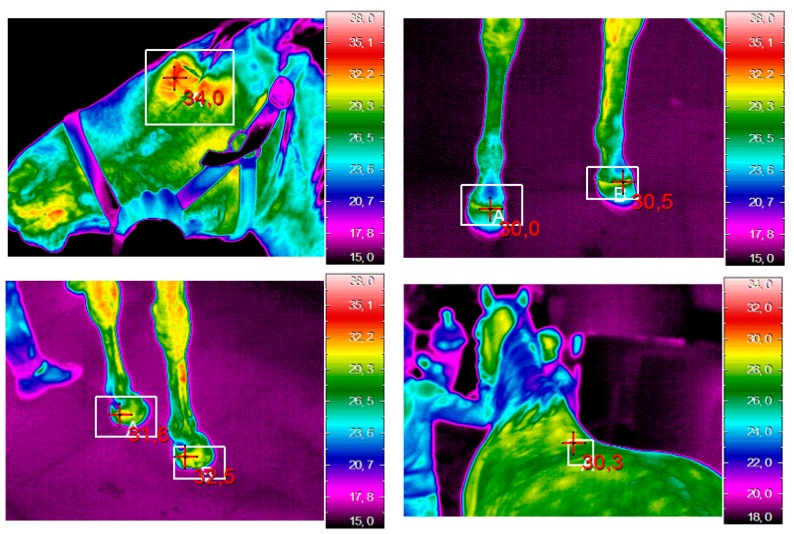
Examples of an infrared image in the examined regions. The cross indicates the position of the maximum temperature within the examined areas used for analysis.

Briefly, BT check was carried out one hour after the morning feeding and immediately before training. During the trial all experimental procedures were sequenced according to invasiveness and possible impact on animal welfare. Experimental procedures were conducted in the following order at each sampling point: HR measurement, IRT and blood sampling. Heart rate was measured by auscultation via stethoscope (Master Cardiology, 3M Litmann, Germany) by the farm veterinarian for a period of 60 s. Thermographic videos were recorded by an infrared camera model Thermo GEAR G120EX (Nippon Avionics Co., Ltd., Tokyo, Japan), with a 320 × 240 uncooled microbolometer sensor. This camera has a thermal sensitivity of 0.04 °C and can detect temperatures ranging from −40 °C to 1500 °C. Images were recorded by two co-authors with experience in IRT (VR, FL) at a frequency of 10 frames/s focusing on the following points: lacrimal caruncle of right/left eye (right/left ET), front right/left crown, rear right/left crown, front right/left pastern, rear right/left pastern, *gluteus* and *longissimus dorsi* muscle (Figure 5). The IRT procedures of each horse started soon after the recording of HR and took about 2 min. While the IR operators moved around the horse quickly, the veterinarian moved to record the HRs, and the other horses’ blood samples were taken by the veterinarian as soon as the HR was recorded and the IRT completed. Overall, the checking procedures started immediately after the training (the time to bring them to the allocated area) and took less than 4 min per horse and were completed within 12 min from the end of the training session.

During the three training sessions, a weather tracker (Kestrel: 4000 Pocket Weather Tracker, Nielsen-Kellerman Co., Boothwyn, PA, USA) was placed inside the cover area adjacent to the walker at horse head height and the following climate parameters were recorded: temperature (°C), humidity (%), atmospheric pressure (mbar), wind speed (km/h), wind direction, and solar radiation (W/m^2^). Ambient temperature and relative humidity values were entered into the camera settings to allow for atmospheric changes during the sampling period, as previously recommended [20]. The two co-authors expert in IRT analyzed all the recorded frame and picked the best shot recording the maximal temperature for each area, as common practice in IRT [13,15,21].

Blood samples were collected into plain and EDTA-Vacutainer tubes (Becton Dickinson, Franklin Lakes, NJ, USA) by jugular venipuncture before and after training by the farm veterinarian. After collection, blood samples were kept at 4 °C and analyzed within 4 h from collection. Routine hematology was determined by a commercial laboratory (HeCo Vet C. Radim Diagnostics, Florence, Italy) using standard laboratory processes and equipment (Cell Dyn 3700 Cell Counter, Abbott, Chicago, IL, USA; and Konelab 20XT photometer, Thermo Fisher Scientific, Vantaa, Finland). Cortisol concentration was assessed in serum samples by immunoassay ELISA technique using a commercial kit (Cortisol Enzyme linked immunosorbent assay kit; art. CEA462Ge, Cloud Clone Corp, Katy, TX, USA). Samples were aliquoted into wells in duplicate and absorbance measured using a wavelength of 405 nm in a microplate plate reader (Multiskan EX, LabSystem, Thermo Fisher Scientific, Milan, Italy).

### 2.2. Statistical Analysis

Statistical analysis was performed using the statistical package SAS (SAS v. 9.4, SAS, Cary, NC, USA). Initially, descriptive statistics was performed, and normality was checked using the Anderson–Darling test. Although all data were normally distributed, some blood parameters were not in the normal range for horses, the observations of those horses were consequently deleted from the dataset. The remaining data were then analyzed by mixed linear model using PROC mixed procedure including time (before and after training, BT and AT), intensity of exercise (low, moderate, high) and their interaction (time × intensity) as fixed factors, and horse as random factor to account for multiple records per animal. A separate model was developed with the following parameters as the outcome: max temperature in the tested points (right/left ET, front right/left crown, rear right/left crown, front right/left pastern, rear right/left pastern, *gluteus* and *longissimus dorsi* muscle), HR, white blood cells (WBC), red blood cells (RBC), hemoglobin (Hgb), hematocrit (Hct), and cortisol level. Comparisons between least squares mean was performed using the Tukey’s test. Pearson correlations among dependent variables were also calculated. For all statistical analyses, a *p* value of <0.05 was considered significant. Results are expressed as least squares mean ± standard error of the means (SE).

## 3. Results

Average environmental parameters recorded during the three training sessions are reported in Table 2.

Table 3 shows the descriptive statistics of the parameters measured, with means, standard deviation, standard error, percent coefficients of variation, and minimum and maximum observed values. The descriptive statistics arranged by intensity and time is shown in Table S1 and Table S2, respectively.

The obtained correlations are reported in Table S3. Heart rate correlated positively with ET (left: *r* = 0.42, *p* = 0.0115; right: *r* = 0.48, *p* = 0.0038), WBC (*r* = 0.58, *p* = 0.0002), RBC (*r* = 0.54, *p* = 0.0006), Hgb (*r* = 0.50, *p* = 0.0015), and Hct (*r* = 0.55, *p* = 0.0005).

Left ET correlated positively with right ET (*r* = 0.81, *p* < 0.001), and with the *gluteus* and *longissimus dorsi* temperature (*r* = 0.50 and *r* = 0.60, *p* = 0.005, and *p* < 0.001, respectively). Similarly, right ET correlated with the same parameters, but also with the temperature registered at both frontal and back crowns (*p* = 0.01). The temperature recorded in both front crowns correlated positively with all the temperature registered in the remaining points (*p* < 0.001), and with cortisol levels (*p* < 0.01) but there was no correlation with the hematological parameters. The temperature registered on the *gluteus* correlated positively with the temperature registered on the *longissimus dorsi* and both temperatures correlated positively with the WBC. No significant correlations were found between WBC and RBC, Hgb, and Hct, while the latter three correlated positively among themselves.

While the interaction time × intensity was never significant, Table 4 shows the effects of time, and intensity on the parameters measured. All the measured parameters were significantly affected by time, showing an increase after training. Heart rate, WBC, and the maximal temperature registered by IRT at the front right and left crown, the front right pastern, and *longissimus dorsi* were significantly affected by intensity, but only HR increased with the increase of training intensity. The other studied parameters were not affected by intensity. Figures S2–S7 show the variations within the same horse across the three bouts (i.e., low, moderate, and high intensity) of the parameters significantly affected by intensity.

## 4. Discussion

This pilot study documented the effects of endurance training at different intensities on HR, blood count, cortisol, and maximal temperature registered by IRT in different points in horses and investigated possible associations among them. The results partially supported our hypothesis. As expected, all the studied parameters increased after the training and ET correlated positively with HR. We also found for the first time that crown temperature (CT) correlated positively with serum cortisol. However, none of the maximal temperatures measured with IRT increased with the intensity of the exercise. Our preliminary findings suggest that IRT, in particular ET and CT may become a useful non-invasive tool to determine the physiological stress caused by endurance training. Further studies should be conducted at the check points during endurance competitions, to determine whether ET and CT may assist veterinarians in deciding which horses are not fit to continue to compete. Overall, IRT may be used to evaluate the effectiveness of a training regime and to understand how horses are coping with the level of physiological stress induced by the different types of training. Infrared thermography technique would be also useful for identifying overtraining and training-related pathologies and injuries and for preventing prolonged suffering of exhausted horses in competitions. Infrared thermography technique would therefore enhance endurance horse welfare, if it were used during training and competition seasons.

The effect of time (before and after training) significantly influenced all variables considered. These results were expected as it is well known that both aerobic and anaerobic physical exercises lead to an increase of HR, cortisol, WBC, RBC, Hbg, Hct, and temperature in horses [22,23,24,25]. The recorded changes of HR and the other hematological parameters were minimal; this may be due to two main reasons, namely the type of training and the fitness of the horses. Our horses were indeed subjected to an aerobic and prolonged exercise but without riders and their social hierarchy was respected, therefore was less physically and psychologically demanding [26]. It is well known that submaximal endurance exercise leads to minimal hematological and metabolic changes [27]. It has been reported that the onset of fatigue would occur after a Thoroughbred horse has cantered while ridden by a jockey over a total distance of 22 km and heavy hunter horse over a distance of 11 km [27,28]. Our horses were exercised over a distance of approximately 20, 30, and 60 km on the three different days but they were not ridden and were trained to keep their gait with the minimal effort. It is also well established that training without a rider is less demanding than training with a rider, for both physical and psychological reasons; there is a significant difference in the results of standard exercise testing for assessing fitness conducted in field and on the treadmill in French trotters [29], or between a riding and a lounging test in sport horse [30]. Consequently, the reported changes were in line with an aerobic, sub-maximal exercise, which made the horse fit without undergoing strenuous exercise under saddle which may lead to higher risk of showing stress-related behaviors and of developing stress-related pathologies, affecting equine health and welfare. Our horses were also in good health and fitness status; the good fitness was confirmed by the very low values of HR recorded AT. Results obtained underlie a good training program, which allowed the animals to cope with the maximum workload. They came back with HR under 64 beats/min within 5 min after exercise termination as requested by FEI regulation during competition [1]. The changes in the maximal temperature measured with IRT also highlighted the value of the training. The changes in the maximal temperature of the eye and of the *longissimus dorsi* were minimal, while the other changes were greater, without reaching levels of concern, which would be typical of inflammation and lameness [6]. Further studies are needed to confirm our findings, testing the animals during maximal exercise and competitions, monitoring HR, HRV, and other physiological parameters, such as respiratory rate and rectal temperature, continuously and in real time using new technological tools such as wearable systems applied to the horses [31].

The intensity of the training was significant only for a few of the studied parameters. However, only HR and WBC increased proportionally to the intensity of the training. Heart rate and WBC are indeed valid indicators to determine the effects of exercise and other stressful events in horses [23,29,30]. Some of the maximal temperatures measured with IRT were affected by the intensity, but the results are not linear. It is indeed worth noting that during our second day the horses trained for longer duration but at a lower speed than the first and the third day, following the typical training plan of the horses. Speed in fieldwork is hard to standardize [29], but it is a crucial factor in equine exercise physiology. Our horses were trained at fixed speed and duration, but we used only eight horses, which showed large difference among them. These latter considerations should be taken in mind while interpreting our data, which need to be confirmed using a larger number of horses and during other standardized exercises with increasing intensity, with both speed and duration increased day by day.

Endurance horses are subjected to very demanding competitions, from both physical and emotional points of view, which may lead to pathologies. The prevalence of stress related pathologies, such as equine gastric ulceration syndrome (EGUS), in high-level endurance horses was indeed of 93% during competition season and 48% during the inter-seasons [32]. Thus, it is important to monitor stress levels both in training and in competition to enhance the health and welfare of the horses used for endurance. In our study ET correlated with HR but not with cortisol. The correlation between ET and HR is in line with the literature [14,16,17] and their correlation could be due to the fact that these two variables seem to have similar physiological bases and they respond rapidly to both physical and emotional stress [4]. This is the reason why ET was used successfully for assessing fear in a novel test [18]. The relation between ET and cortisol seems instead to be more complex and the results in the literature are conflicting. Eye temperature correlated well to both salivary and plasma cortisol induced by an injection of adrenocorticotropic hormones [33], but Valera et al. [15] did not find any correlations between ET and salivary cortisol after a show jumping competition. In our study cortisol increased AT but it was not affected by the intensity of the training; it did not correlate with ET, while it correlated with CT. The latter finding is new and deserves further attention. The CT values have been proposed as a possible indicator for laminitis [9], which can be induced by high levels of cortisol in horses. The relationship between ET, CT, and cortisol, their possible different responses, and their timing in response to a stressor are complex and deserve further examination. It would be interesting to observe not only the variation in BT and AT, but also the trend of increasing ET and CT measured with IRT during physical effort, in order to better evaluate the reliability of ET and CT for assessing stress in performance horses.

Our findings need to be interpreted with caution since this was a pilot study with a small number of horses used. The interaction between time and treatment was never significant, probably due to the small sample size and the minimal difference in intensity between the first and the second day tested. Another limitation of this pilot study was that a number of parameters critical to exercise physiology and exercise stress such as rectal temperature, lactate, and muscle enzymes levels were not measured or recorded in this pilot study. Consequently, all the findings need to be ascertained in future studies using more horses, in order to minimize the individual variability, recording more parameters and testing after maximal exercise or competitions. It is indeed worth highlighting that our findings were collected during sub-maximal exercises and would be valid only for similar exercises. Finally, even though we tried to keep the emotional stress very low, respecting the horse routine and training in group without riders, in this study it was impossible to evaluate whether the recorded changes where caused—and in what proportion—by physical or emotional stress. Notwithstanding those limitations, this study reached its aim of increasing our knowledge on the effects of different types of endurance training on IRT and other stress indicators. Based on the correlations found between HR and ET, this study suggests that ET and CT may be a useful tool to identify horses suffering from high levels of physiological stress during endurance competitions.

## 5. Conclusions

This study investigated the effects of different intensities of endurance training on physiological stress indicators. The study suggests that the IRT seems to be a good indicator of physiological stress and may be useful to evaluate the effectiveness of a training in endurance horses. Based on our preliminary findings, ET was confirmed to be an indicator of physical and emotional stress, while IRT registered in the other regions seems to be more related to thermoregulation and the increase in blood flow due to exercise. Further studies are needed during actual competition to understand whether ET and CT may become a useful non-invasive tool to identify exhausted horses and withdraw them from the competition to prevent serious implications for their health and welfare.

## Figures and Tables

**Table 1:** Details of the horses included in the trial, with their birth year, sex (G stands for gelding and F for female), coat color, and number of previous competitions.

Horse	Year	Sex	Coat	Total Competitions
BR	2008	G	Bay	6
BC	2008	G	Bay	5
AR	2007	F	Bay	9
BT	2007	F	Gray	8
TA	2007	F	Bay	7
FD	2007	G	Gray	13
OV	2001	G	Gray	19
ZR	2006	F	Gray	11

**Table 2:** Average values of the environmental parameters recorded during the three training sessions (low, moderate, high intensity).

Parameters	Low Intensity Training Day	Moderate Intensity Training Day	High Intensity Training Day
Humidity (%)	67%	92%	83%
Temperature (°C)	17.2	16.0	17.2
Atmospheric pressure (mbar)	1022	1017	1013
Wind speed (km/h)	7.5	6.5	2.5
Wind direction	East	East	East
Rain (mL)	0	0	0
Solar Radiation (W/m^2^)	3.0	0.2	0.2

**Table 3:** Descriptive statistics of the parameters measured with means, standard deviations (SDs), percent coefficients of variation (%CV), standard errors (SEs), and minimum (Min) and maximum (Max) observed values.

Parameters	Mean	SD	%CV	%CV *	SE	Min	Max
HR (bpm)	49.8	9.8	19.6	19.0	1.6	32.0	64.0
Cortisol (ng/mL)	115.2	9.4	8.1	8.4	1.4	86.6	128.3
Right ET(°C)	34.6	0.7	2.1	2.0	0.1	32.4	36.1
Left ET (°C)	34.4	0.8	2.4	2.4	0.1	32.2	36
Front right crown (°C)	28	3.8	13.4	14.1	0.6	20.3	32.2
Front left crown (°C)	28.2	3.5	12.4	13.1	0.5	20.8	32.3
Rear right crown (°C)	27.7	3.8	13.6	14.3	0.6	17.5	34.5
Rear left crown (°C)	27.8	3.7	13.2	13.9	0.6	18.0	31.6
Front right pastern (°C)	29.7	4.0	13.4	14.2	0.6	20.4	34.0
Front left pastern (°C)	29.9	3.8	12.8	13.4	0.6	20.3	34.1
Rear right pastern (°C)	29.7	4.1	13.7	14.8	0.6	17.9	35.2
Rear left pastern (°C)	29.5	3.9	13.1	14.0	0.6	17.8	33.3
*Gluteus* (°C)	31.8	2.3	7.4	7.4	0.4	26.1	36.8
*Longissimum dorsi* (°C)	31.3	1.8	5.8	6.0	0.3	26.8	33.8
WBC (k/μL)	10.6	2.6	24.5	21.4	0.4	5.4	18.6
RBC (M/μL)	8.5	0.9	11.1	10.6	0.1	6.8	10.9
Hgb (g/dL)	15.6	1.9	12.4	12.1	0.3	12.2	21.5
Hct (%)	33.1	3.8	11.4	10.6	0.6	25.5	42.2

* Percent coefficient of variation calculated after removing the observations of one horse with parameters outside the normal limits.

**Table 4:** Effects of time and intensity on heart rate, serum cortisol level, and smaximal temperature recorded in different points, and white blood cells (WBCs), red blood cells (RBCs), hemoglobin (Hgb), and hematocrit (Hct).

Parameters	Time			Intensity				*p*-Value	
	BT	AT	SE	Low	Moderate	High	SE	Time	Intensity
HR (bpm)	41.8	55.5	1.2	45.3 ^a^	46.4 ^a^	55.2 ^b^	1.8	<0.0001	0.0016
Cortisol (ng/mL)	111.4	119.0	3.0	114.2	116.1	115.2	3.1	0.0010	0.7599
Right ET(°C)	34.2	34.9	0.2	34.7	34.4	34.3	0.2	0.0049	0.2020
Left ET (°C)	34.1	34.7	0.2	34.7	34.1	34.4	0.2	0.0056	0.0585
Front right crown (°C)	25.3	30.4	0.6	27.7 ^a,b^	29.3 ^b^	26.5 ^a^	0.7	<0.0001	0.0242
Front left crown (°C)	25.9	30.2	0.5	27.7 ^a^	29.8 ^b^	26.5 ^a^	0.6	<0.0001	0.0022
Rear right crown (°C)	25.1	30.0	0.6	27.2	28.7	26.9	0.7	<0.0001	0.2242
Rear left crown (°C)	25.2	30.0	0.6	27.2	29.1	26.7	0.7	<0.0001	0.0830
Front right pastern (°C)	27.2	32.0	0.6	29.3 ^a,b^	31.3 ^b^	28.1 ^a^	0.7	<0.0001	0.0105
Front left pastern (°C)	27.6	32.0	0.7	29.7	31.1	28.7	0.8	<0.0001	0.0800
Rear right pastern (°C)	27.4	31.8	0.8	29.3	31.1	28.5	0.9	<0.0001	0.0897
Rear left pastern (°C)	27.4	31.6	0.8	29.1	31.1	28.3	0.9	0.0002	0.0643
*Gluteus* (°C)	30.0	33.5	0.4	31.4	32.0	31.8	0.4	<0.0001	0.4070
*Longissimus dorsi* (°C)	30.0	32.5	0.4	31.9 ^b^	30.7 ^a^	31.2 ^b^	0.4	<0.0001	0.0404
WBC (k/μL)	9.3	11.2	0.7	9.9 ^a^	9.8 ^a^	11.1 ^b^	0.7	<0.0001	0.0142
RBC (M/μL)	8.4	8.7	0.3	8.7	8.3	8.6	0.3	0.1424	0.2089
Hgb (g/dL)	15.4	16.0	0.5	15.8	15.2	16.0	0.5	0.2714	0.3761
Hct (%)	32.5	34.0	0.9	34.2	32.1	33.3	1.0	0.0989	0.1673

^a,b^*p* < 0.05.

## Supplementary Materials

The following are available online at https://www.mdpi.com/2076-2615/9/3/84/s1, Figure S1: Photo of the walker/horse training machine from above, Figure S2: Heart rate (HR) variations within the same horse across the 3 bouts: low (1), moderate (2), and high (3) intensity, Figure S3: Front right crown temperature variations (expressed in °C) within the same horse across the 3 bouts: low (1), moderate (2), and high (3) intensity, Figure S4: Front left crown temperature variations (expressed in °C) within the same horse across the 3 bouts: low (1), moderate (2), and high (3) intensity, Figure S5: Front right pastern temperature variations (expressed in °C) within the same horse across the 3 bouts: low (1), moderate (2), and high (3) intensity, Figure S6: *Longissimus dorsi* temperature variations (expressed in °C) within the same horse across the 3 bouts: low (1), moderate (2), and high (3) intensity, Figure S7: White blood cell (WBC) variations (expressed in k/μl) within the same horse across the 3 bouts: low (1), moderate (2), and high (3) intensity, Table S1: Descriptive statistics of the studied parameters expressed by intensity, Table S2: Descriptive statistics of the studied parameters expressed by time, Table S3: Pearson correlations among the studied parameters.
