# Supplementary material for: The Use of Infrared Thermography (IRT) as Stress Indicator in Horses Trained for Endurance: A Pilot Study
Source: Animals (Basel). 2019 Mar 7;9(3):84. doi: 10.3390/ani9030084 (PMC6466296; doi:10.3390/ani9030084)
Supplement: Supplementary file 1 [file animals-09-00084-s001.zip › animals-433382-sup3/Table S1,S2, Figure S1-S7.docx]

**Supplementary Materials**

**Table S1**. Descriptive statistics of the studied parameters expressed by intensity, with means, standard deviation (SD), standard error (SE) and minimum (Min) and maximum (Max) values.

|  | **Low intensity** | | | | |  | **Moderate intensity** | | | | |  | **High intensity** | | | | |
| --- | --- | --- | --- | --- | --- | --- | --- | --- | --- | --- | --- | --- | --- | --- | --- | --- | --- |
| **Parameters** | **Mean** | **SD** | **SE** | **Min** | **Max** |  | **Mean** | **SD** | **SE** | **Min** | **Max** |  | **Mean** | **SD** | **SE** | **Min** | **Max** |
| HR (bpm) | 46.7 | 8.2 | 2.4 | 32.0 | 57.0 |  | 46.4 | 8.8 | 2.4 | 33.0 | 63.0 |  | 58.2 | 8.0 | 2.5 | 43.0 | 64.0 |
| Cortisol (ng/ml) | 114.9 | 8.0 | 2.1 | 102.6 | 124.4 |  | 116.1 | 9.8 | 2.6 | 90.9 | 128.3 |  | 114.8 | 10.8 | 2.9 | 86.6 | 124.2 |
| Left ET (°C) | 34.7 | 0.6 | 0.1 | 33.7 | 36.0 |  | 34.1 | 0.8 | 0.2 | 32.2 | 34.9 |  | 34.5 | 1.0 | 0.3 | 32.3 | 35.7 |
| Right ET (°C) | 34.8 | 0.5 | 0.1 | 33.8 | 35.8 |  | 34.5 | 0.7 | 0.2 | 33.1 | 35.6 |  | 34.4 | 1.0 | 0.3 | 32.4 | 36.1 |
| Front Left Crown (°C) | 28.3 | 3.3 | 0.8 | 20.8 | 31.3 |  | 29.8 | 1.2 | 0.3 | 28 | 31.6 |  | 26.2 | 4.5 | 1.2 | 20.8 | 32.3 |
| Front Right Crown (°C) | 28.3 | 3.6 | 0.9 | 20.4 | 31.7 |  | 29.4 | 1.8 | 0.5 | 24.3 | 31.3 |  | 26.2 | 4.8 | 1.3 | 20.3 | 32.2 |
| Rear Left Crown (°C) | 27.6 | 3.8 | 1.0 | 19.3 | 31.1 |  | 29.1 | 2.0 | 0.5 | 23.4 | 30.8 |  | 26.6 | 4.5 | 1.2 | 18.0 | 31.6 |
| Rear right Crown (°C) | 27.7 | 3.6 | 0.9 | 19.5 | 32.2 |  | 28.9 | 2 | 0.5 | 23.1 | 31.6 |  | 26.7 | 5.0 | 1.3 | 17.5 | 34.5 |
| Front Left Pastern (°C) | 30.3 | 3.8 | 0.9 | 20.3 | 34.0 |  | 31.1 | 1.5 | 0.4 | 28.4 | 33.1 |  | 28.3 | 5.0 | 1.4 | 21.7 | 34.1 |
| Front Right Pastern (°C) | 29.9 | 3.9 | 1.0 | 20.4 | 34.0 |  | 31.4 | 1.5 | 0.4 | 28.5 | 33.5 |  | 27.7 | 5.0 | 1.4 | 20.7 | 33.5 |
| Rear Left Pastern (°C) | 29.5 | 4.3 | 1.1 | 18.1 | 33.3 |  | 30.9 | 1.3 | 0.4 | 28.2 | 33.0 |  | 28 | 4.6 | 1.3 | 17.8 | 32.2 |
| Rear Right Pastern (°C) | 29.9 | 4.4 | 1.1 | 18.8 | 35.2 |  | 31.0 | 1.9 | 0.5 | 26.1 | 33.4 |  | 28.3 | 4.9 | 1.3 | 17.9 | 34.6 |
| Gluteal Femoral (°C) | 31.6 | 2.0 | 0.5 | 27.9 | 34.5 |  | 32.1 | 1.5 | 0.4 | 29 | 34.3 |  | 31.7 | 3.4 | 0.9 | 26.1 | 36.8 |
| *Longissimus dorsi* (°C) | 32.0 | 1.3 | 0.3 | 29.5 | 33.8 |  | 30.7 | 1.7 | 0.5 | 27.9 | 33.2 |  | 30.9 | 2.2 | 0.6 | 26.8 | 33.8 |
| WBC (k/μl) | 10.5 | 1.9 | 0.5 | 8.5 | 14.7 |  | 9.8 | 2.6 | 0.7 | 5.4 | 15.1 |  | 11.3 | 3.2 | 0.9 | 7.2 | 18.6 |
| RBC (M/μl) | 8.5 | 1.0 | 0.2 | 6.8 | 9.9 |  | 8.3 | 0.9 | 0.2 | 7.1 | 10.2 |  | 8.7 | 0.9 | 0.2 | 7.6 | 10.9 |
| Hgb (g/dl) | 15.4 | 1.9 | 0.5 | 12.2 | 18.1 |  | 15.2 | 1.7 | 0.5 | 12.9 | 18.6 |  | 16.3 | 2.1 | 0.6 | 13.5 | 21.5 |
| Hct (%) | 33.2 | 3.9 | 1.0 | 25.5 | 38.2 |  | 32.2 | 3.8 | 1.0 | 27.2 | 38.9 |  | 33.8 | 3.7 | 1.0 | 29.2 | 42.2 |

**Table S2**. Descriptive statistics of the studied parameters expressed by time, with means, standard deviation (SD), standard error (SE) and minimum (Min) and maximum (Max) values.

|  | **Before training (BT)** | | | | |  | **After training (AT)** | | | | |
| --- | --- | --- | --- | --- | --- | --- | --- | --- | --- | --- | --- |
| **Parameters** | **Mean** | **SD** | **SE** | **Min** | **Max** |  | **Mean** | **SD** | **SE** | **Min** | **Max** |
| HR (bpm) | 40.8 | 7.2 | 1.9 | 32.0 | 60.0 |  | 55.5 | 6.2 | 1.3 | 44.0 | 64.0 |
| Cortisol (ng/ml) | 111.8 | 10.9 | 2.4 | 86.6 | 124.4 |  | 118.7 | 6.0 | 1.3 | 107.7 | 128.3 |
| Left ET (°C) | 34.1 | 0.8 | 0.2 | 32.2 | 35.4 |  | 34.8 | 0.7 | 0.2 | 32.7 | 36.0 |
| Right ET (°C) | 34.3 | 0.7 | 0.1 | 32.4 | 35.4 |  | 34.9 | 0.6 | 0.1 | 33.5 | 36.1 |
| Front Left Crown (°C) | 26.3 | 3.8 | 0.8 | 20.8 | 31.6 |  | 30.1 | 1.4 | 0.3 | 26.9 | 32.3 |
| Front Right Crown (°C) | 25.7 | 4.0 | 0.8 | 20.3 | 31.3 |  | 30.4 | 1.2 | 0.3 | 28 | 32.2 |
| Rear Left Crown (°C) | 25.7 | 4.0 | 0.9 | 18.0 | 31.1 |  | 30.0 | 1.1 | 0.2 | 26.9 | 31.6 |
| Rear right Crown (°C) | 25.6 | 4.0 | 0.8 | 17.5 | 30.7 |  | 30.0 | 1.5 | 0.3 | 28.1 | 34.5 |
| Front Left Pastern (°C) | 27.8 | 4.2 | 0.9 | 20.3 | 32.4 |  | 32.1 | 1.7 | 0.4 | 28.4 | 34.1 |
| Front Right Pastern (°C) | 27.3 | 4.4 | 1.0 | 20.4 | 32.7 |  | 32.0 | 1.4 | 0.3 | 28.5 | 34.0 |
| Rear Left Pastern (°C) | 27.5 | 4.6 | 1.0 | 17.8 | 33.0 |  | 31.5 | 1.0 | 0.2 | 29.6 | 33.3 |
| Rear Right Pastern (°C) | 27.6 | 4.8 | 1.0 | 17.9 | 32.8 |  | 31.6 | 1.8 | 0.4 | 26.1 | 35.2 |
| Gluteal Femoral (°C) | 30.0 | 1.5 | 0.3 | 26.1 | 32.7 |  | 33.6 | 1.5 | 0.3 | 30.9 | 36.8 |
| *Longissimus dorsi* (°C) | 30.2 | 1.5 | 0.3 | 26.8 | 32.4 |  | 32.4 | 1.3 | 0.3 | 28.8 | 33.8 |
| WBC (k/μl) | 9.6 | 2.0 | 0.4 | 5.4 | 13.9 |  | 11.5 | 2.8 | 0.6 | 6.3 | 18.6 |
| RBC (M/μl) | 8.2 | 1.0 | 0.2 | 6.8 | 10.9 |  | 8.7 | 0.8 | 0.2 | 7.6 | 10.2 |
| Hgb (g/dl) | 15.2 | 2.1 | 0.4 | 12.2 | 21.5 |  | 16.1 | 1.7 | 0.4 | 13.5 | 18.6 |
| Hct (%) | 31.9 | 3.9 | 0.8 | 25.5 | 42.2 |  | 34.2 | 3.3 | 0.7 | 29.1 | 38.9 |

**Figure S1**. Photo of the walker/ horse training machine from above.

**Figure S2**. Heart rate (HR) variations within the same horse across the 3 bouts: low (1), moderate (2) and high (3) intensity. HR is expressed in BPM. Error bars represent the standard deviation of the repeated measures for each horse.

**Figure S3**. Front right crown temperature variations (expressed in °C) within the same horse across the 3 bouts: low (1), moderate (2) and high (3) intensity. Error bars represent the standard deviation of the repeated measures for each horse.

**Figure S4**. Front left crown temperature variations (expressed in °C) within the same horse across the 3 bouts: low (1), moderate (2) and high (3) intensity. Error bars represent the standard deviation of the repeated measures for each horse.

**Figure S5**. Front right pastern temperature variations (expressed in °C) within the same horse across the 3 bouts: low (1), moderate (2) and high (3) intensity. Error bars represent the standard deviation of the repeated measures for each horse.

**Figure S6**: *Longissimus dorsi* temperature variations (expressed in °C) within the same horse across the 3 bouts: low (1), moderate (2) and high (3) intensity. Error bars represent the standard deviation of the repeated measures for each horse.

**Figure S7**. White blood cell (WBC) variations (expressed in k/μl) within the same horse across the 3 bouts: low (1), moderate (2) and high (3) intensity. Error bars represent the standard deviation of the repeated measures for each horse.
